# Cell Cycle Deregulation in Ewing's Sarcoma Pathogenesis

**DOI:** 10.1155/2011/598704

**Published:** 2010-11-01

**Authors:** Ashley A. Kowalewski, R. Lor Randall, Stephen L. Lessnick

**Affiliations:** ^1^Department of Oncological Sciences, University of Utah School of Medicine, 2000 Circle of Hope, Room 4242, Salt Lake City, UT 84112, USA; ^2^Center for Children's Cancer Research, Huntsman Cancer Institute, University of Utah School of Medicine, 2000 Circle of Hope, Room 4242, Salt Lake City, UT 84112, USA; ^3^Sarcoma Services, Department of Orthopaedics, University of Utah School of Medicine, 2000 Circle of Hope, Room 4242, Salt Lake City, UT 84112, USA; ^4^Division of Pediatric Hematology/Oncology, University of Utah School of Medicine, 2000 Circle of Hope, Room 4242, Salt Lake City, UT 84112, USA

## Abstract

Ewing's sarcoma is a highly aggressive pediatric tumor of bone that usually contains the characteristic chromosomal translocation t(11;22)(q24;q12). This translocation encodes the oncogenic fusion protein EWS/FLI, which acts as an aberrant transcription factor to deregulate target genes necessary for oncogenesis. One key feature of oncogenic transformation is dysregulation of cell cycle control. It is therefore likely that EWS/FLI and other cooperating mutations in Ewing's sarcoma modulate the cell cycle to facilitate tumorigenesis. This paper will summarize current published data associated with deregulation of the cell cycle in Ewing's sarcoma and highlight important questions that remain to be answered.

## 1. Introduction

First described by James Ewing in 1921, Ewing's sarcoma is characterized as a highly aggressive, undifferentiated tumor of the bone [1]. Although it is the second most common primary bone tumor in children and adolescents, Ewing's sarcoma can also develop in extraosseous sites as a soft-tissue malignancy [2, 3]. The etiology of Ewing's sarcoma remains unknown, but there appears to be a predominance of cases within the Caucasian population, with males being slightly more susceptible than females [3, 4]. This disease is highly invasive with approximately one-fourth of all Ewing's sarcoma patients presenting with metastases at the time of diagnosis [2, 5]. Current treatment methods include surgery, radiation, and systemic chemotherapy [6]. Despite such an aggressive regimen, the 5-year disease-free survival rate for patients with localized Ewing's sarcoma is only 60–70% and that for individuals presenting with metastases drops to a mere 30% [5, 7].

Approximately 85% of Ewing's sarcoma tumors harbor the reciprocal translocation t(11;22)(q24;q12), which fuses the 5′ portion of *EWSR1* from chromosome 22 with the 3′ portion of *FLI1* from chromosome 11 [8, 9]. *EWSR1* encodes the EWS protein, which belongs to the TLS/EWS/TAF15 (TET) family of putative RNA-binding proteins [10, 11]. Understanding the physiologic roles of TET proteins has recently become of greater scientific interest as data continues to surface identifying these members as being intrinsic to the development of other sarcomas arising from similar chromosomal translocations. Currently, EWS has been hypothesized to perform a number of functions, including, but not limited to: RNA transcription and/or processing, neuronal cell differentiation, meiosis, B-lymphocyte development, and proneural cell survival in the developing zebrafish embryo [12]. Interestingly, it also appears that EWS may play an important role in mitotic integrity, which will be discussed in more detail later [13]. 

*FLI1 *encodes FLI, a member of the ETS family of transcription factors [14]. Wild-type FLI participates in hematopoietic, vascular, and neural crest development [15–17]. FLI contains an 85-amino-acid domain in its carboxy-terminus which mediates sequence-specific DNA binding [8, 17, 18]. This consensus *ets* domain recognizes the conserved core sequence motif GGAA/T, with bases flanking the core sequence contributing to affinity and specificity [9, 19]. A total of 27 ETS family members have been identified in the human genome [17]. 

The (11;22) chromosomal translocation gives rise to the fusion protein EWS/FLI. This protein product pairs the DNA-binding domain of FLI with a strong transcriptional activation domain from EWS, thereby generating an aberrant transcription factor [14, 18]. Many genes have been identified that are regulated by EWS/FLI, some of which have been shown to be necessary for the development of Ewing's sarcoma [20–28]. Interestingly, recent data suggests that a significant percentage of deregulated genes are indirect targets of EWS/FLI, reinforcing the long-held belief that EWS/FLI-mediated oncogenesis likely involves both direct and indirect mechanisms of targeted gene deregulation [19]. 

Defects in the regulation of normal cell proliferation are characteristic of all transformed cells [29]. Mutations affecting genes involved in networks regulating cell cycle often underlie such uncontrolled proliferation, which subsequently becomes exploited during oncogenesis [30, 31]. Previous data has shown that EWS/FLI is an oncogene. Therefore, it is likely to mediate alterations in cell cycle, either alone or in concert with mutations in other genes, to control cell proliferation in Ewing's sarcoma. Recently, data published by Kauer et al. has lent credence to this belief. Specifically, the authors demonstrated through the development of a molecular function map of Ewing's sarcoma that a large number of EWS/FLI upregulated genes participate in regulation of the cell cycle [32]. Importantly, these data were generated using both primary patient-derived cell lines as well as primary tumor samples obtained from individuals with Ewing's sarcoma, suggesting that these results are correlative with the disease process *in vivo*. Consequently, a better understanding of how the cell cycle is deregulated by EWS/FLI to support uncontrolled cell proliferation will provide critical insight into its overall role in tumorigenesis.

One major issue that has made studying EWS/FLI-mediated cell cycle deregulation difficult, however, is the fact that the cell of origin for Ewing's sarcoma is unknown. Consequently, investigators have used various heterologous systems to determine how EWS/FLI may impinge on the cell cycle in order to facilitate oncogenesis. Unfortunately, an inherent problem with this approach is that different cell types exhibit different levels of tolerance to EWS/FLI expression. For instance, NIH-3T3 cells expressing EWS/FLI become transformed whereas YAL-7 cells, a derivative of NIH-3T3 cells, fail to do so [33]. In addition, expression of EWS/FLI in primary murine fibroblasts results in cell death whereas immortalized human fibroblasts undergo a p53-dependent growth arrest with concomitant downregulation of several cell cycle regulators [33, 34]. Further confounding the issue is that comparisons of gene expression profiles derived from a number of microarray data sets suggested that heterologous systems do not always recapitulate the gene expression of human tumors [35, 36]. To circumvent this problem, many groups have started using patient-derived Ewing's sarcoma cell lines [17, 21–23, 28, 32, 37–40]. Loss of EWS/FLI expression in many of these cell lines results in growth inhibitory effects [20, 37, 41–44]. Interestingly, however, there does exist one cell line that does not exhibit such effects, A673. Although initially characterized erroneously as a rhabdomyosarcoma cell line, A673 cells have been shown to express EWS/FLI and recapitulate the gene expression pattern of *bona fide* Ewing's sarcoma [25, 36]. Loss of EWS/FLI expression in A673 cells does not inhibit their proliferation *in vitro* [25, 45]. Consequently, the use of this particular cell line to study EWS/FLI-mediated transformation has allowed changes in cell cycle regulation specific to the oncogenic process to be identified. By understanding the interplay between EWS/FLI and regulators of cell cycle one may be able to determine why such discrepancies in tolerance are seen between different cell lines and may lead to the identification of specific conditions permissive to the development of Ewing's sarcoma.

## 2. General Cell Cycle

Cell cycle progression is a highly regulated process. Defects in the cell cycle machinery can undermine this regulation, subsequently leading to uncontrolled cell proliferation as well as genomic and chromosomal instability, all of which are a prelude to oncogenesis [46, 47]. There are four different phases of the cell cycle, which can be grouped into two general processes: interphase and mitosis [48]. Interphase consists of three distinct phases, referred to as G1, S, and G2, which are involved in cellular growth and replication of the genome. During mitosis, or the M phase, cells undergo the process of division, which culminates in the generation of two identical daughter cells. As cells exit from mitosis, they can either re-enter the cell cycle at the G1 phase or be diverted to the G0 phase where they enter into a quiescent state. 

**Figure 1:**
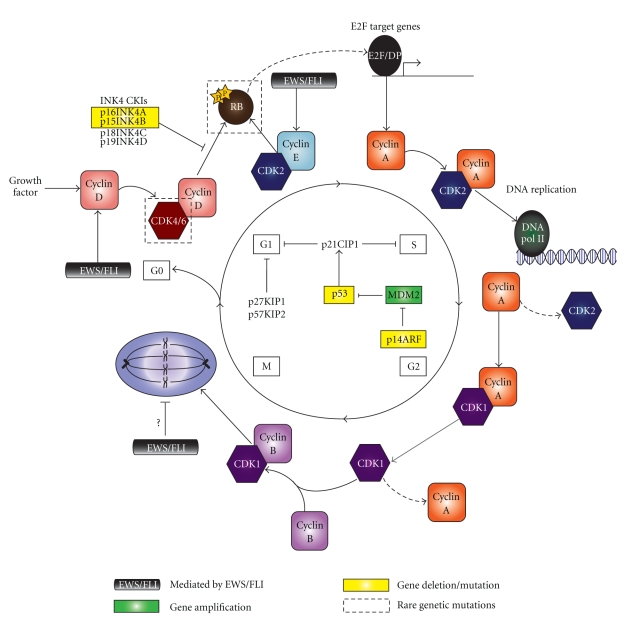
Overview of the cell cycle. Progression through the cell cycle is mediated by cyclin-CDK complexes that regulate RB function. Cyclin-dependent kinase inhibitors negatively regulate this process in order to control DNA replication and cell division. Based on the data presented above, regulators of the cell cycle thought to be involved in Ewing's sarcoma pathogenesis are indicated. (Note: in an effort to be concise, only the most significant regulators and their respective functions are included in the diagram).

Cyclin-dependent kinases, or CDKs, are the master regulators of the cell cycle, and it is defects in their function that often lead to inappropriate cell growth [46, 47]. CDKs are serine/threonine kinases that, when bound to their respective cyclin subunits, become activated and phosphorylate RB [49]. This leads to the dissociation of the transcription factor E2F from RB, which then allows for the transactivation of target genes necessary for cell cycle progression. CDK activity is controlled in multiple ways: the availability of cyclin subunits, the presence of posttranslational modifications, and the existence of cyclin-dependent kinase inhibitors, or CKIs [50]. CKIs play an important inhibitory role in the cell cycle as they can bind to and repress cyclin-CDK activity when necessary. Cyclins and CKIs themselves are regulated by changes in the rate of gene expression and proteolysis throughout the cell cycle in order to maintain proper progression. The intricacies of the cell cycle will be discussed in more detail in the following sections, along with current data to suggest how EWS/FLI contributes to its deregulation in facilitating the development of Ewing's sarcoma (see Figure 1 for an overview of the cell cycle). 

## 3. The G1 and S Phases of Cell Cycle

G1 may arguably be the most important phase of the cell cycle as it is the first line of defense against oncogenic transformation. Multiple levels exist within the G1 phase to ensure that cells harboring mutations or other detrimental aberrations are not allowed to enter S phase. This is extremely important as once cells have passed through the restriction (R) point into S phase, they are irreversibly committed to completing that particular round of cell cycle [48]. The RB pathway plays an essential role in this process, as it is the tightly regulated phosphorylation of RB itself that progression through G1 and into S phase is controlled. 

The retinoblastoma gene *RB1 *encodes the protein product RB, which has a molecular weight of approximately 110 kDa [51]. Two other members of the RB family of proteins, p107 and p130, function similarly to RB, and their expression/role in cell cycle tends to be cell and context dependent [52]. It is the phosphorylation status, which is regulated by extracellular cues, of RB that determines whether cells progress through the cell cycle. In the presence of growth factors, mitogenic signaling activates the expression of D- and E-type cyclins. There are three different D-type cyclins (i.e., cyclins D1, D2, and D3) and two different E-type cyclins (i.e., cyclins E1 and E2), all of which exhibit different tissue-specific patterns of expression [30, 48, 49, 53]. Cyclin D proteins interact with the cyclin-dependent kinase CDK4 (or its isoform CDK6 in certain cell types) whereas cyclin E proteins interact with CDK2, both of which act as allosteric activators to generate functional kinase complexes. Initially, the cyclin D-CDK4 complex targets RB for phosphorylation [54]. Once this has occurred, RB then becomes a substrate for cyclin E-CDK2. It is following a second round of phosphorylation by cyclin E-CDK2 that RB dissociates from the transcription factor E2F, subsequently facilitating progression through G1 and into S phase of the cell cycle.

Surprisingly, mutations targeting RB itself are extremely rare in primary Ewing's tumors, as are mutations involving *CDK4 *gene amplification and/or overexpression of cyclin D1 [49, 55–61]. However, experiments analyzing expression of cell cycle regulators in the presence and absence of EWS/FLI have provided additional information as to the mechanisms being utilized during the development of Ewing's sarcoma. For instance, the level of EWS/FLI tends to correlate directly with the amount of cyclin D1 being expressed [43, 61]. Interestingly, EWS/FLI appears to control even what isoform of cyclin D1 is present (i.e., cyclin D1b), favoring that which has been shown to be more oncogenic [40]. Moreover, EWS/FLI also appears to modulate the level of cyclin E proteins in order to facilitate accelerated proliferation, an attribute previously unidentified from tumor sample analyses [62, 63]. Although it seems that EWS/FLI controls proliferation by deregulating G1 phase cell cycle regulators, one must be cautious in interpreting such results. These data were generated using patient-derived cell lines that exhibit growth arrest following inhibition of EWS/FLI. As previously discussed, not all cell lines undergo growth arrest or change in growth rate with changes in EWS/FLI expression [25]. Importantly, changes in cyclin levels have not been consistently observed in such cell lines. Thus, it is not clear whether a direct cause-effect relationship between EWS/FLI and cyclin expression exists, or whether such correlations are due instead to growth effects mediated through other EWS/FLI-dependent pathways. 

The activity of CDK4/6 is negatively regulated by a set of cyclin-dependent kinase inhibitors belonging to the INK4 family of proteins (a second CKI family is involved in the regulation of CDK2, which will be discussed in more depth later in the paper). There are four members of the INK4 family, which include INK4A (or p16), INK4B (or p15), INK4C (or p18), and INK4D (or p19) [31, 48, 57]. These CKIs are small proteins that act to inhibit kinase activity in two ways: (1) by binding to CDK4/6, preventing association with D-type cyclins and (2) by interacting with the catalytic cleft of CDK4/6, thus inhibiting the binding of ATP molecules required for the phosphorylation of its substrates [64]. Consequently, the presence of INK4 CKIs results in the inability of CDK4/6 to phosphorylate RB, thus inhibiting progression in the G1 phase of the cell cycle.

The most frequent genetic aberration associated with Ewing's sarcoma involves deletion of the *CDKN2A* locus, which results in functional loss of the p16 gene [59]. Interestingly, the gene encoding p15 (*CDKN2B*) maps to the same chromosomal region as *CDKN2A*, and so deletions present in cancer cells involving *CDKN2A* often also affect *CDKN2B*, resulting in functional loss of both p16 and p15. Approximately 10–30% of all primary Ewing's tumors exhibit either homozygous or hemizygous deletion of the *CDKN2A* gene and sometimes also deletion of the *CDKN2B* gene [10, 49, 55, 57, 65–67]. Importantly, a number of analyses have shown that individuals with this particular mutation exhibit a statistically significant correlation with poor overall survival, but not with metastatic presence or chemotherapeutic response [60, 68, 69]. 

One interesting characteristic of Ewing's tumors is that the frequencies of genetic aberrations described above tend to be much different in patient-derived primary Ewing's sarcoma cell lines. This is evidenced by the fact that more than 50% of all cell lines appear to exhibit loss of functional p16. Deletions of the *CDKN2A* locus as well as hypermethylation of the gene promoter both appear to contribute to this type of mutation with the latter being almost nonexistent in primary tumor samples [10, 57]. It is not entirely understood why such a discrepancy exists, but the fact that deletion of *CDKN2A* is the most prevalent mutation associated with Ewing's sarcoma suggests an important role for the alteration in at least a subset of Ewing's sarcoma. 

Following dissociation of the transcription factor E2F from its inhibitory interaction with RB, expression of target genes required for cells to progress through S phase occurs. Included in this is the expression of A-type cyclins [30, 48]. There are two different A-type cyclins, cyclin A1 and cyclin A2 [70, 71]. Cyclin A1 expression is restricted to cells in the developing embryo as well as those undergoing meiosis. However, cyclin A2 is ubiquitously expressed in all proliferating somatic cells, thus acting as the main A-type cyclin involved in cell cycle processes. Cyclin A2 associates with CDK2 and activates its kinase function, subsequently facilitating the phosphorylation of target proteins required for S phase progression. Most importantly, the cyclin A2-CDK2 complex has been shown to play a vital role in the process of DNA replication [72]. Interestingly, no studies to date have identified mutations or alterations involving cyclin A2 or CDK2 that could contribute to the process of Ewing's sarcoma oncogenesis.

## 4. The G2 and M Phases of Cell Cycle

Following the completion of DNA replication, cells exit S phase and enter the G2 phase of cell cycle. At this point, cyclin A2 becomes associated with a second cyclin-dependent kinase, CDK1 (also referred to as CDC2) [31, 50]. The role of cyclin A2-CDK1 is to facilitate the transition from G2 to the mitotic phase of the cell cycle, specifically by regulating the activity and degradation of proteins involved in this process [73]. Regulation at this transition point in the cell cycle ensures that only cells with a full complement of intact DNA are allowed to divide. Absence of such regulation can result in oncogenesis via any number of mechanisms, including the deletion of a single gene, parts of or whole chromosomes, and even aberrant chromosomal fusions, as is seen with Ewing's sarcoma [48].

In order to progress through mitosis, cells rely on the presence of B-type cyclins. There are three different family members, including cyclins B1, B2, and B3. Cyclin B3 is expressed mainly in meiotic cells in embryonic and adult tissues whereas cyclins B1 and B2 are more generally expressed in somatic cells [74]. In addition, cyclin B2 localizes to the Golgi while cyclin B1 is found mainly within the nucleus, thus acting as the major B-type cyclin involved in mitosis. At the beginning of prophase, cyclin B1-CDK1 complexes form in order to phosphorylate (and in some cases activate) proteins involved in the progression of mitosis [75]. In order for cells to enter anaphase, CDK1 and cyclin B1 are degraded, followed by completion of cell division. At this point, cells are able to exit mitosis, thus facilitating a second round of cell cycle. 

Currently there are no published studies to suggest that cyclins or CDKs involved in G2 and M phase of the cell cycle are deregulated in primary Ewing's tumor samples. However, a few studies have identified other components of the pathway that EWS/FLI may target to control the cell cycle. For example, EWS/FLI appears to upregulate expression of the murine homolog of human EZ-C/UbcH10, a cyclin-specific ubiquitin conjugating enzyme [76]. Increased levels of this protein were shown to enhance the destruction of mitotic cyclins as well as inhibit CDK1 activity, thus promoting the onset of anaphase and mitotic exit. Consequently, cells are able to progress through mitosis more quickly and to continually re-enter the cell cycle, thus resulting in continued proliferation. Lack of regulation during mitosis can lead to oncogenesis as checkpoints normally present in the cell that act to prevent aberrant exit, and re-entry may cause cells with missing/damaged DNA to continue to proliferate.

Recently it has been suggested that wild-type EWS plays an important role in mitotic spindle formation and that the presence of EWS/FLI interferes with this function by acting in a dominant-negative fashion [13]. Specifically, Embree et al. demonstrated using both a zebrafish model system as well as HeLa cells that ectopic expression of EWS/FLI results in cells exhibiting gross mitotic spindle defects. Interestingly, loss of wild-type EWS produced the same phenotype. Further experiments showed that abnormal localization of aurora kinase B preceded the presence of spindle defects in cells, suggesting that under normal conditions wild-type EWS plays a role in facilitating proper localization of aurora kinase B during mitosis. Another group has also suggested that aurora kinases A and B are transcriptionally modulated by EWS/FLI [77]. Additional data is needed, however, to show that disruption of mitotic spindle formation is a means by which EWS/FLI modulates the cell cycle in the pathogenesis of Ewing's sarcoma.

## 5. Broad Regulators of Cell Cycle

### 5.1. The p53-ARF-MDM2 Pathway

In conjunction with the CDK4/6 inhibitor p16INK4A, mutations of the *TP53* gene are the most recurrent genetic alterations associated with cancer [49]. The *TP53* gene encodes the well-known tumor-suppressor p53, which plays an important role in various regulatory processes involved with cell cycle progression. Most importantly, p53 acts as a negative regulator, facilitating growth arrest, senescence, and/or apoptosis when cells are exposed to genotoxic, cytotoxic, and/or physiologic stresses [78, 79]. p53 is a transcription factor possessing two transcriptional activation domains and a DNA-binding domain that recognizes specific sequences. Following exposure to cellular stress, p53 activates expression of *CDKN1A*, which encodes the cyclin-dependent inhibitor p21CIP1. It is through the expression of p21 (and other transcriptional targets) that p53 negatively regulates the cell cycle (a more detailed discussion of this particular CKI and the family of proteins it belongs to will be provided in the next section) [64, 80–82].

The expression level and activity of p53 is regulated by two different proteins: MDM2 and p14ARF. MDM2 is an E3 ubiquitin ligase, which facilitates the degradation of p53 in a ubiquitin- and proteasome-dependent manner [83]. Interestingly, *MDM2* is a transcriptional target of p53, whose expression is increased in concert with increased levels of p53 [84]. In addition to promoting degradation, MDM2 can also inhibit p53 function through a direct protein-protein interaction, suppressing its transcriptional activity, as well as translation of the mRNA transcript itself. In order to combat these negative regulatory effects, p14ARF is able to positively regulate p53, in part through the negative regulation of MDM2. p14ARF is actually found at the same genetic locus as *CDKN2A,* which encodes the CKI p16 [39, 84]. The two gene sequences overlap but possess alternate reading frames and are independently regulated. ARF binds MDM2, preventing it from interacting with p53 [78]. Consequently, p53 becomes stabilized, and its overall activity increases within the cell [67]. 

Although more than half of all primary patient-derived Ewing's sarcoma cell lines possess direct p53 loss-of-function mutations, only about 5–20% of Ewing's tumors exhibit similar genetic defects [10, 19, 49, 57, 66, 67, 81, 83, 85]. Yet, many of these studies have shown that patients harboring p53 mutations not only have a significantly poorer outcome with respect to both disease-free and overall survival but also have a higher probability of relapse [66, 81, 86–88]. Mutations in MDM2 seem to be extremely rare in Ewing's tumors [10, 56, 60]. However, since a large percentage of Ewing's tumors exhibit deletion of the *CDKN2A* locus, it is likely that they also harbor deletions in p14ARF [89]. Consequently, targeted disruption of *TP53 *may not be as important for the pathogenesis of Ewing's sarcoma as is general inhibition of the p53 pathway.

Identifying genetic aberrations present in both tumor samples and cell lines may provide information necessary to understand how EWS/FLI mediates Ewing's sarcoma oncogenesis. One important example of this is the discrepancy seen with respect to p53 loss-of-function mutations. Previous experiments have shown that increased expression of EWS/FLI results in an increase in p53 expression, as well as an increased apoptotic response following irradiation [90]. In addition, ectopic expression of the fusion protein in various primary cell lines, including human foreskin fibroblasts, has been shown to cause p53-mediated growth arrest [34]. These results indicate that in order for EWS/FLI to induce transformation, the p53 pathway must be deregulated in some way. Thus, it is likely that primary tumors expressing wild-type p53 harbor other genetic abnormalities yet to be identified that impinge on other aspects of the p53 pathway. Understanding how the p53 pathway is modulated in Ewing's sarcoma may help to increase the efficacy of therapies used to combat the malignancy as most chemotherapy and radiation treatments induce cancer cell death in a p53-dependent manner [79]. 

### 5.2. The CIP/KIP Family of CKIs

As was discussed earlier, there is a second family of cyclin-dependent kinase inhibitors, referred to as the CIP/KIP family of proteins [30]. There are three different members: p21CIP1, p27KIP1, and p57KIP2. Similar to the INK4 family of CKIs, CIP/KIP proteins inhibit the kinase activity of CDKs by preventing their association with cyclin subunits and ATP molecules, both of which are required for the phosphorylation of target substrates [48, 64]. Unlike the INK4 family, however, CIP/KIP proteins are able to functionally inhibit multiple CDKs. For instance, both p27 and p57 can inhibit the kinase activity of CDK4/6 and CDK2 whereas p21 acts to control the function of both CDK2 as well as CDK1 [31, 80]. Of the three family members, p21 is the most diverse, performing a variety of functions in addition to controlling CDK activity. For example, it was previously mentioned that p21 is a transcriptional target of p53 [64, 80–82, 91]. Following exposure to various stresses, p53 activates the expression of p21, thus contributing to a DNA damage response [80]. In addition, p21 has been shown to localize to the cytoplasm where it acts to inhibit the induction of apoptosis by interacting with proteins involved in mediating this process. 

Most of the data acquired to date regarding the role of CIP/KIP proteins in Ewing's sarcoma oncogenesis have come from experiments using cell lines. However, a few pieces of *in vivo* data do exist that corroborate the *in vitro* findings. For instance, loss of p21 and p27 expression has been seen in both formalin-fixed paraffin-embedded (FFPE) and primary tumor samples [58, 62, 92]. Importantly, FFPE samples exhibiting decreased levels of p21 and p27 came from patients that exhibited a significantly shorter overall rate of survival. Interestingly, however, similar results were not seen with respect to the primary tumor samples analyzed. In addition, EWS/FLI has been shown to inhibit the expression of these two proteins in various cell lines, with EWS/FLI levels correlating inversely with those of p21 and p27 [43, 44, 93]. Furthermore, while one group has demonstrated that p21 may be a direct transcriptional target of EWS/FLI, identification of an *in vivo* binding site has thus far alluded investigators [94]. Unlike p21, however, there is currently no data to suggest that p27 is a direct target of EWS/FLI-mediated deregulation. Surprisingly, very little data exists to suggest that p57 contributes to the oncogenic process of Ewing's sarcoma. Yet, one group did identify that expression of EWS/FLI correlates inversely with p57 expression similar to that found for p21 and p27 [38]. Consequently, it is possible that genetic alterations targeting these CIP/KIP family members could act as a mechanism for EWS/FLI-mediated transformation in the development of Ewing's sarcoma. However, further studies are required to understand the biological significance of these results.

### 5.3. Additional Regulators

The last two cell cycle regulators that will be discussed in this paper are C-MYC and Ki67. Both of these proteins play crucial roles during cell proliferation but do so in very different ways. C-MYC influences several processes involved in cell cycle regulation via its function as a transcription factor. For example, C-MYC, when bound to its partner MAX, has been shown to induce the expression of cyclins D1 and D2 as well as CDK4, subsequently promoting G1 phase progression [48, 95]. The MYC-MAX heterodimer can also support continued cell cycle progression through the repression of multiple CKIs, including p15, p18, p21, and p27 [96]. Furthermore, C-MYC can increase the expression of E2F2 and cyclin A2, both of which effect the S phase of cell cycle and contribute to overall proliferation [96–98]. Similar to C-MYC, Ki67 has been shown to be vital for cell proliferation [99]. Ki67 is a cell proliferation-associated nuclear antigen that is thought to contribute to cell cycle progression via its involvement in rRNA and ribosome synthesis [99, 100]. Interestingly, Ki67 is expressed in all of the phases of cell cycle (excluding G0), but whether or not it participates in other such related processes is currently unknown.

Directly or indirectly, C-MYC activity appears to be deregulated in almost all tumor cells [95]. Experiments using primary patient-derived Ewing's sarcoma cell lines have indicated that C-MYC is a gene target of EWS/FLI [38, 93, 101, 102]. However, efforts to identify a *bona fide* EWS/FLI binding site have proved unsuccessful. Expression levels of the fusion protein appear to correlate directly with those of C-MYC, resulting in an overall increase in cell proliferation. Nonetheless, this conclusion is somewhat controversial, as experiments demonstrating this relationship are found only to exist under conditions where loss of EWS/FLI expression inhibits cell growth, raising the question of whether this result is limited to particular experimental conditions or is specific to the disease process. Interestingly, it has also been shown that EWS/FLI expression increases the levels of ID2, or inhibitor of DNA binding 2. ID2 is the dominant-negative form of a basic helix-loop-helix transcription factor, which lacks a functional DNA-binding domain. ID2 contributes to C-MYC-induced cell proliferation by interacting with RB, ultimately suppressing its ability to inhibit cell cycle progression. Primary tumor samples also appear to exhibit increased ID2 expression, but the biological implications of these results with respect to the development of Ewing's sarcoma are currently unknown.

Intriguingly, primary Ewing tumor samples have been shown to exhibit a significant correlation between C-MYC expression and the percent of cells positive for Ki67, which acts as a measure of overall proliferation [67, 103]. Patients exhibiting a higher Ki67 positivity tended to have a better chemotherapeutic response, as measured by the amount of tumor necrosis following initial treatment [87]. However there are conflicting results as to whether or not Ki67 positivity correlates with a worse prognosis [103]. Ki67 positivity is used as both a prognostic and diagnostic tool for other malignancies, however, suggesting that further studies should be conducted in order to determine if such use is also applicable to Ewing's sarcoma [99]. 

## 6. Conclusion

Ewing's sarcoma is a highly aggressive disease with a relatively low survival rate. Determining how deregulation of the cell cycle contributes to the oncogenic process has led to an increased understanding of Ewing's sarcoma development (see Figure 1). However, many questions remain unanswered. For instance, does a single mutation explain deregulation of the cell cycle in Ewing's sarcoma? The most common genetic alteration associated with Ewing's tumors involves deletion of *CDKN2A*, which encodes the p16 and p14 proteins. Yet, only about one-third of all tumors actually possess such mutations. In addition, the second most frequent aberration identified to be associated with Ewing's sarcoma involves mutation of *TP53*. Again, however, less than one-fourth of all Ewing's tumors actually possess a mutant form of p53. The remaining genetic alterations found to facilitate deregulation of the cell cycle in Ewing's sarcoma are even rarer. Moreover, differences in mutational frequencies between primary tumors and cell lines have further complicated the issue of identifying genetic alterations specific to the disease process. Consequently, no single mutation has been identified that appears to specifically mediate deregulation of the cell cycle in Ewing's sarcoma.

 Although no individual mutation has been identified to explain how the cell cycle is deregulated in Ewing's sarcoma, most tumors contain genetic alterations that specifically affect the activity of cyclin-dependent kinase inhibitors. For instance, mutations in *CDKN2A *and *CDKN2B* result in functional loss of p16 and p15, respectively. In addition, deactivating mutations in p14ARF (often resulting from deletions in *CDKN2A*) and p53 as well as amplification of *MDM2* all lead to functional loss of p21. Those mutations affecting positive cell cycle regulators such as cyclins and CDKs occur much less frequently and often have only been identified in *in vitro* experiments using patient-derived cell lines. Consequently, it is possible that underlying Ewing's sarcoma development is the inactivation of CKIs, a common mechanism by which the cell cycle is deregulated.

Since no common mechanism of cell cycle deregulation in Ewing's sarcoma has been identified, it raises the question of whether or not EWS/FLI contributes to its deregulation. Currently there is no clear answer, as most EWS/FLI-mediated changes in cell cycle regulation have been identified solely under particular experimental conditions where inhibition of EWS/FLI expression elicits growth arrest. However, it is important to point out that EWS/FLI must have some effect on the cell cycle since previous experiments have shown that mutations in specific regulators (such as p53) are often required to create a permissive environment for EWS/FLI expression. In addition, some type of mutation affecting cell cycle regulation is present in a large percentage of primary Ewing's tumors analyzed, suggesting that EWS/FLI activity does trigger cell cycle inhibitory pathways and that these pathways must be bypassed prior to tumor development. Thus, although not fully understood, it does appear that EWS/FLI contributes to uncontrolled cell proliferation by affecting pathways involved in cell cycle control. 

Identification of EWS/FLI as being necessary for the development of this malignancy has provided great insight into some of the molecular mechanisms that underlie the oncogenic process. However, despite the fact that EWS/FLI is necessary for Ewing's sarcoma pathogenesis, other cooperating mutations are required. Such cooperating mutations appear to target components of the cell cycle in order to facilitate uncontrolled cell proliferation. The different genetic aberrations currently known have been summarized above. However, there is no single genetic alteration yet discovered that would suggest a specific mechanism being employed during tumor development. Future experiments attempting to understand how cell cycle deregulation contributes to the oncogenesis of Ewing's sarcoma will hopefully provide greater insight into the disease process, thus leading to more efficacious treatment therapies that can be used to combat this disease.
